# MAFB in Pancreatic β-Cell Development and Dysfunction: Implications for Diabetes and Translational Applications

**DOI:** 10.3390/biom16081076

**Published:** 2026-07-23

**Authors:** Razik Bin Abdul Mu-u-min, Abdoulaye Diane, Heba Hussain Al-Siddiqi

**Affiliations:** Diabetes Research Center, Qatar Biomedical Research Institute (QBRI), Hamad Bin Khalifa University (HBKU), Qatar Foundation (QF), Doha P.O. Box 34110, Qatar

**Keywords:** MafB, β-cell, diabetes, β-cell identity, β-cell function

## Abstract

Large MAF transcription factors, including MafA and MafB, are essential for maintaining β-cell identity, function and survival. While MafA has been widely studied in pancreas development and type 2 diabetes, the extended roles of MafB in humans are still emerging. During embryogenesis, MafB promotes differentiation of β-cells. While MafB is downregulated in adult mouse β-cells, it remains active in adult human β-cells, indicating important species-specific functions. Mechanistically, MafB cooperates with other β-cell-specific transcription factors, including PDX1, NEUROD1 and NKX6.1, to regulate genes critical for β-cell differentiation and insulin expression. Loss of MafB in human β-cells is associated with hallmark features of diabetic pathology, such as dedifferentiation, impaired insulin production, and transdifferentiation under metabolic stress. In addition to its endocrine roles within islets, MafB regulates macrophage polarization and apoptotic cell clearance, suggesting immune–metabolic interactions that may contribute to islet inflammation and dysfunction. Translationally, MafB may be leveraged to enhance stem-cell-derived β-cell differentiation and maturation and support β-cell identity preservation under stress and potentially immune responses; however, these applications remain to be further studied and validated. In this review, we integrate findings from developmental biology, animal models, and human studies to clarify the overarching role of MafB in bridging β-cell development, immune regulation, and potential translational application in stem-cell therapy or as a biomarker target in diabetes research.

## 1. Introduction

Diabetes mellitus refers to a group of chronic metabolic disorders characterized by persistent elevated blood glucose levels. The main mechanisms underlining the disease are insulin resistance and β-cell dysfunction, both of which result in impaired glucose metabolism. The International Diabetes Federation (IDF) reported that, as of 2024, 589 million people live with diabetes, with a 46% increase expected by 2050 (www.idf.org/about-diabetes/diabetes-facts-figures/ (accessed on 1 January 2026). The two major types of diabetes are type 1 diabetes (T1D), characterized by immune-mediated destruction of pancreatic β-cells causing a near complete deficiency of insulin production, and type 2 diabetes (T2D), wherein individuals develop insulin resistance and insulin deficiency due to β-cell dysfunction [1,2,3,4,5]. A central pathological theme of both T1D and T2D is the dysfunction of pancreatic β-cells, which negatively affects insulin secretion. Understanding the key transcriptional regulators that govern β-cell identity, function and resistance to stress is critical to advancing therapeutic strategies targeting β-cell preservation or replacement.

Among the numerous transcription factors implicated in β-cell biology, members of the large MAF family, in particular MafA and MafB, have attracted prominent attention. These transcription factors bind to MAF recognition elements (MAREs) in the regulatory regions of target genes (e.g., PDX1, insulin) and activate gene expression programs essential for β-cell differentiation and function [6]. While MafA has been well-characterized for its role in mature β-cells, especially in mice, MafB’s role is still emerging as a distinct regulator of β-cell function as well as survival [7,8,9].

Experimental evidence suggests that MafB is essential during development of insulin-producing β-cells, with key roles in lineage commitment and early endocrine maturation [10]. Notably, MafB continues to be expressed in adult human β-cells, unlike in mice where its expression is downregulated postnatally, suggesting species-specific regulatory roles [11,12]. Loss of MafB expression has been observed in diabetic islets, implicating its role in β-cell identity loss under metabolic stress [13].

In this review, we propose that MafB is a critical regulator of β-cell development and function and a key determinant of β-cell health and survival. We consolidate the current knowledge on MafB, focusing on its expression profile and regulatory function in pancreatic β-cells and its role in β-cell dysfunction, immune-mediated responses and diabetes pathogenesis. By compiling findings from mouse models, human islet studies, and in vitro systems, we aim to describe the underlying mechanisms through which MafB influences β-cell health and dysfunction and to evaluate its clinical relevance as a potential biomarker and a molecular target for translational research in diabetes.

## 2. Musculoaponeurotic Fibrosarcoma B (MafB)

**Figure 1:**
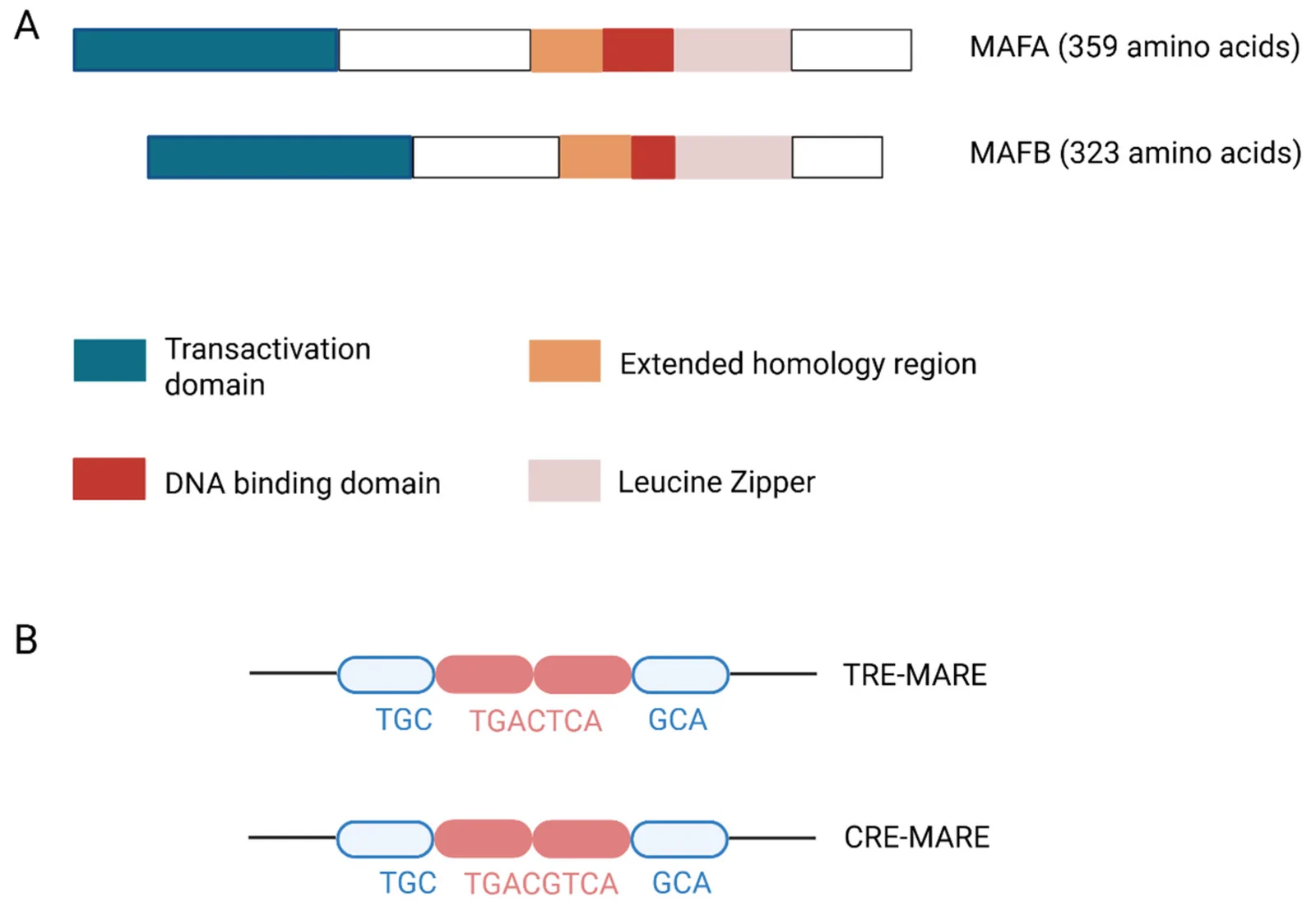
(**A**). Schematic figure of MafA and MafB protein structures. Key structural domains include the transactivation domain, extended homology region, DNA binding domain and the leucine zipper region [21]. (**B**). Consensus sequences recognized by bZip transcription factors like MafA and MafB. TRE and CRE (pink) form the core MARE sequence flanked by three bases on either side (blue), which assists in recognition of the MARE by MAF proteins [22]. Created in BioRender. Al-Siddiqi, H. (2026) https://BioRender.com/093lajf (accessed on 10 July 2026).

The musculoaponeurotic fibrosarcoma (MAF) family consists of regulatory functional genes that can be classified into two types: small MAFs without an N-terminal transactivation domain (e.g., MafF, MafG and MafK) and large MAFs (e.g., MafA, MafB, c-Maf and NRL) [14]. MAFs function as transcription factors and play important developmental and regulatory roles in the pancreas, eyes, cartilage, and other tissues [15,16,17]. MafB, belonging to the large MAF classification, contains a transactivation domain in its N-terminus and a basic leucine zipper domain (bZIP) at its C-terminus, containing an extended homology region, a basic region for DNA contact and a leucine zipper for dimerization. Like other large MAF proteins, MafB recognizes a DNA consensus region known as MAF recognition elements (MAREs), which include a 5′ TGC sequence flanking an AP-1-like core motif. The MAF extended homology region helps it contact the TGC flanking region, while the basic region allows them to bind to TPA responsive elements (TREs) or cAMP responsive elements (CREs) (Figure 1) [6]. MafB has the ability to homodimerize and bind MARE sequences with high specificity, and it also forms heterodimers with v-MAF and Fos proteins, thereby broadening its regulatory function. Thus, MafB facilitates DNA binding and the formation of homo/heterodimers with other bZIP transcription factors [18]. In HeLa cells co-transfected with MafB gene, it was observed that MafB plays an important cooperative role with transcription factors such as PDX1 and ISL1 to enhance insulin activation more than MafA, which is another known MAF transcription factor involved in β-cell development and function by regulating insulin transcription in mature β-cells [14]. MafB can also recruit chromatin-modifying methyltransferase coregulator complexes mixed-lineage leukemia 3 and 4 (MLL3/4) to alter chromatin structure and gene expression, thereby regulating β-cell function [19]. MafB has also been shown to bind ETS-1 though its zipper-binding domain and can repress erythroid lineage commitment [20]. Taken together, MafB holds promise to function as a context-dependent transcriptional regulator across multiple tissues.

## 3. Role of MafB in β-Cell Development and Function

MafB is a key transcription factor that plays a significant role in pancreatic endocrine development and β-cell maturation, with functions that are highly context-dependent across developmental stages and species. In mice, MafB is strongly expressed during the development of pancreatic endocrine cells, including both insulin-producing β-cells and glucagon-producing α-cells [15,23]. During rodent pancreas development, hormone-producing cells appear in waves: the first glucagon-producing cells appear around embryonic day E9–9.5, followed by the appearance of insulin-producing cells at embryonic day E11.5. A secondary transition occurs at E13.5 and is marked by the appearance of the second wave of proliferating endocrine cells, mainly β-cells [24,25,26]. MafB is expressed both in α- and β-cells at E10.5 and E12.5, respectively, and is found in most insulin-positive cells at E14.5–E18.5 [7,23]. The transcriptional regulation of MafB in pancreas development is linked to the hierarchy of endocrine developmental factors. Ngn3 (neurogenin-3), a key endocrine transcription factor expressed in pancreatic progenitors, activates a cascade of genes, such as NEUROD1, PAX4, PAX6, NKX2.2, and ARX, among others, which specify each endocrine subtype. PAX6 is particularly important for islet cell differentiation (α- and β-cells), and in mice, Pax6 deficiency reduced the number of MafB^+^ cells formed. The authors observed that MafB deficiency does not affect the initiation of endocrine differentiation, suggesting a downstream role for MafB in the pancreatic differentiation cascade, thereby contributing primarily to the maturation rather than the specification of endocrine cell lineages [27].

At the molecular level, MafB acts as a key transcriptional activator in the embryonic β-cell gene network and directly binds to MARE sequences in the promoters/enhancers of key β-cell genes, such as insulin gene promoter and β-cell transcription factor PDX1, thereby forming a key factor in the basic functioning of a β-cell, which is production and secretion of insulin in response to glucose challenge [8,28]. Gene expression profiling studies at E18.5 between wildtype and *MafB*^−/−^ mice have revealed the presence of thirty-five genes whose mRNA levels were altered by more than 1.5-fold. The candidate genes thus found were genes mainly involved in β-cell maturation and function, such as ion binding and transport (*Slc30a8*, *Rbp4*, *Nnat*, *Slc2a2*), signal transduction (*Tpte2*) and hormone secretion (insulin). Hence, MafB’s activity during this window is essential for β-cells to attain a mature phenotype. In humans, gene-editing experiments reinforce this function of MafB as observed in a human pluripotent stem-cell (hPSC)-derived β-cell differentiation study wherein the MafB gene was not detected until the pancreatic progenitor stage and increased expression was observed only towards the end stage of differentiation. The same study also showed that, even though MafB gene expression was detected at the pancreatic progenitor stage, protein levels of MafB were only detectable at the maturation stage of the differentiated β-like cells, suggesting robust MafB expression coinciding with the onset of hormone expression [28]. The role of MafB becomes prominent at the end stage of differentiation, where cells lacking MafB failed to respond to GSIS, a key function of β-cells, suggesting β-cell dysfunction. Thus, MafB is required in late-stage β-cell differentiation to achieve its mature state, as it prepares β-cells to produce and secrete insulin in adequate amounts [28,29].

**Figure 2:**
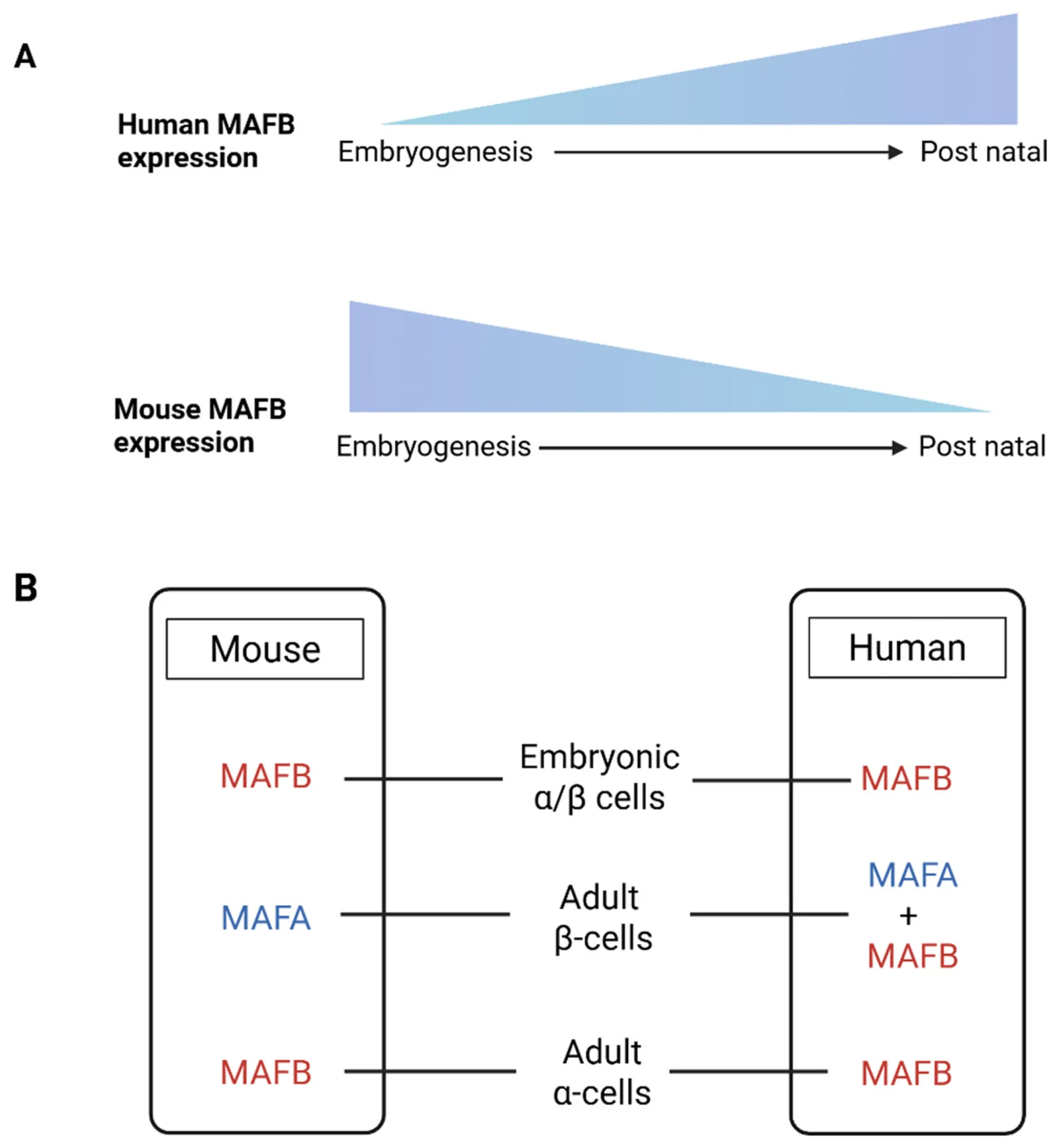
Species specific differences in MafB expression. Conceptual overview of the developmental roles of MafB during pancreatic endocrine differentiation from literature cited in this review. (**A**) In humans, MafB expression is low during early embryogenic stages and increases over time to highest expression postnatally. In sharp contrast, in mice, MafB expression is highest during embryogenesis and is turned off postnatally. (**B**) Key Maf factor in mice and humans at distinct stages of α/β-cell life. MafB plays the primary role in α/β-cell development in mice and humans. While MafA is the primary Maf factor in adult mice β-cells, MafA and MafB play important roles in adult human β-cells. MafB is the primary Maf factor in adult α-cells. Created in BioRender. Al-Siddiqi, H. (2026) https://BioRender.com/y1o432w (accessed on 10 July 2026).

It is noteworthy that MafB has distinct expression patterns in rodents versus primates, suggesting species-specific differences in expression patterns (Figure 2). In mice, postnatally, MafB expression begins to decline in β-cells and is completely depleted by approximately 3 weeks after birth by de novo DNA methylation while maintaining expression in α-cells [7,23,30,31]. Pancreas-wide deletion mutant MafB mice (*MafB^∆Panc^*) showed transient elevated glucose levels after birth (neonatal hyperglycemia), which was resolved by the second week due to the compensatory role of MafA to maintain β-cell function in the absence of MafB. This compensatory mechanism ensures normal insulin secretion upon maturation and highlights the stage-specific roles of Maf factors in rodent islet development. This was confirmed by the generation of pancreas MafA/MafB knockout mice (*MafAB^∆Panc^*), which died shortly after birth due to hyperglycemia and loss of insulin-positive cells [32]. Gene profiling of MafB knockout vs. wildtype mice pancreata at E18.5 suggests a temporal separation of MafA and MafB expression, proposing a sequential model wherein MafB predominantly acts first to play a role in β-cell development, initiating the expression of key genes involved in glucose sensing, vesicle storage, and regulation of fasting glucose levels (e.g., *Slc2a2*, *Slc30a8* (ZnT8), and *G6pc2*) as β-cells are developed, while MafA maintains and enhances the expression of the same genes in adult β-cells. This indicates that, in mice, MafA takes over from MafB to continuously regulate a common set of β-cell maturation genes once cells reach postnatal life. Therefore, MafB ‘primes’ β-cell development, while MafA ‘perpetuates’ β-cell identity and function in adulthood [7]. Although MafA and MafB have overlapping targets, the two transcription factors are not entirely functionally redundant. Loss-of-function studies revealed that MafA loss in adult β-cells cannot be fully compensated by MafB. In MafA-depleted mice, MafB alone could not rescue gene expression changes resulting from the loss of MafA, including *Ins2*, *G6pc2*, *Slc30a8*, and *Slc2a2* [31].

In contrast, MafB is expressed during the developmental stage and postnatal lifespan in human β-cells, which was confirmed at protein and single-cell RNA sequencing studies [12,28,31]. Knockout of MafB in human pluripotent stem cells impairs the generation of insulin-positive and glucagon-positive cells, suggesting that MafB is required for proper α- and β-cell differentiation in humans. In human β-cells, where both MafA and MafB are co-expressed, evidence suggests the presence and function of MafA–MafB heterodimers. Transgenic mouse experiments reported that co-expression of MafB and MafA in adult β-cells caused no obvious phenotype change, implying that the MafA/MafB heterodimer can perform the same transcriptional roles as the normal MafA homodimer in mice. This suggests that human adult β-cells (MafA^+^ and MafB^+^) likely achieve similar gene regulation as mouse β-cells (MafA^+^ only) [12,31]. A recent study has underscored MafA’s and MafB’s role in regulating the insulin exocytosis apparatus of β-cells. In human islets, expression of 29 exocytosis genes positively correlated to MafA and MafB levels. Silencing MafA or MafB in human islets and EndoC-βH1 cells impaired glucose-stimulated insulin secretion (GSIS) and reduced the expression of key exocytosis genes, such as *STX1A*, *STXBP1*, and *SYT7*. Also, islets from T2D donors lacked MafA expression and showed reduced levels of *STX1A* and *STXBP1*. These data indicate that MafA and MafB coregulate a network of β-cell maturation genes responsible for insulin packaging and release [29]. A summary of MafA’s and MafB’s roles in β-cells is given in Table 1.

Extended roles of MafB in pancreatic development

Apart from regulating endocrine-specific genes, MafB also influences developmental processes such as cell migration and islet morphogenesis. A recent study has revealed that MafB controls the expression of certain neurotransmitter receptors and axon guidance molecules in the developing pancreas, leading to proper clustering of endocrine cells into islets. The study observed that the development of a MafB-knockout mouse pancreas was impaired in islet and ductal morphogenesis. 3D optical projection tomography and 2D immunohistochemical analysis of wildtype β-cells showed large clusters around the developing mucin/ductal network at E18.5, while *MafB*^−/−^ knockout islets were located closer to ductal epithelium, along with an impaired ductal network in the knockouts. These effects were not observed at E12.5, suggesting the impairment arose at later stages of pancreatic differentiation. Moreover, gene ontology analysis of *MafB*^−/−^ and wildtype mice endocrine cells showed differentially expressed genes associated with cell migration and synaptic signaling. Key neurotransmitter receptor genes *ChrnA3*, *ChrnA4*, *ChrnB4*, *P2ry1*, and *Adraa2A* were significantly reduced at E14.5 and E15.5, along with important axon guidance receptor genes, such as *Robo1*, *Robo2*, *Nrp1*, and *Nrp2*, among others, suggesting MafB’s role in regulating transcription of axon guidance and neurotransmitter signaling pathways responsible for cell migration and islet formation [33]. During development, adrenergic signaling from pancreatic nerves is important for islet formation [34]. Bsharat et al. observed that β-cell migration and clustering were impaired upon inhibition of nicotinic signaling in co-cultures of embryonic β-cells with autonomic cells, suggesting the role of acetylcholine signaling in islet innervation and formation. The authors linked loss of MafB to impaired nicotinic signaling activity as a possible mechanism of the role of MafB to β-cell clustering and migration to autonomic tissue [33]. MafB is also important for α-cell differentiation and function, underlining its broader role in islet development. In the embryonic pancreas, MafB is expressed in α-cells as well as β-cells. Also, as important as MafB is to β-cell development, MafB performs an equally important function in the developing α-cell by binding to the glucagon gene promoter (at the G1 element found between nucleotides −77 and −51) and is required for appropriate glucagon transcription [23]. Although Conrad et al. showed that selective pancreatic knockdown of MafB did not significantly change glucagon content or α-cell numbers, glucagon secretion was reduced upon exposure to low glucose concentrations and the potent secretagogue arginine. There were also no changes in α-cell-enriched transcription factors (*Arx* and *Brn4*) or other α-cell-enriched gene products (*HigD1a*, *Ndst4*, *Pdk4*) in the MafB pancreatic knockouts. The authors, thus, suggested that MafB controls postnatal α-cell function [32]. Collectively, these studies show that MafB is a multifaceted regulator in pancreatic endocrine lineage. It acts as a transcriptional activator of key pancreatic β-cell genes such as PDX1 and insulin, hence playing important roles in β-cell identity and function, as its loss delays β-cell maturation in mice and GSIS dysfunction in humans. Moreover, their activity extends to islet structural assembly, as well as maturation and function of pancreatic α-cells. Much of the current understanding of MafB function in pancreatic endocrine biology is derived from studies using mouse KO models; human β-cell lines, such as EndoC-β cells; and hPSC-derived β-cells. These systems have provided critical mechanistic insights into transcriptional regulation and developmental pathways. However, each model also presents limitations that must be carefully considered. Interpretation of MafA and MafB function requires careful consideration of species-specific differences. In mice, MafB plays an essential role in β-cell development during embryogenesis but is largely downregulated in adult β-cells, whereas MafA becomes the dominant transcriptional regulator of mature β-cells [9,31]. Consequently, MafB deletion in adult mice does not appear to severely impair β-cell survival, whereas MafA deficiency leads to major defects in insulin secretion and β-cell maturity [32]. In contrast, studies in human endocrine islets indicate that MafB expression persists in adult β-cells, suggesting that MafB retains functional roles beyond development [12,28,31]. This difference raises the possibility that compensatory mechanisms observed in mice models may not fully apply to human islet biology. Therefore, conclusions derived from murine models should be cautiously interpreted when extrapolating to human disease, particularly in the context of β-cell identity and diabetes progression. In addition to the differences in rodent vs. human expression of MafA/MafB, it is also important to note that immortalized cell lines and stem-cell-derived β-like cells may not fully recapitulate the functional and transcriptional complexity of mature human islets [35,36]. Consequently, careful deliberation is warranted when extrapolating the findings from these experimental models to explain human β-cell biology and diabetes pathophysiology. A summary of experimental studies of MafB in rodent and human models with respect to β-cell development and function are presented in Table 2.

**Table 1:** Summary of the key differences between MafA and MafB.

Feature	MafA	MafB
Primary role	Adult β-cell function and identity. Required for mature β-cell maintenance and insulin secretion [7].	Developing β-cell differentiation (and adult α-cells): required for generation of insulin+ and glucagon+ cells [7]. In human β-cells, it contributes to adult β-cell function [28].
Species differences (rodents vs. humans)	Rodents: MafA is expressed in adult β-cells but not in α-cells. Humans: Largely absent in fetal/juvenile β-cells and appears only ~9 years of age [12].	Rodents: MafB is co-expressed in developing α- and β-cells and then gets restricted to mature α-cells. Humans: MafB is expressed in both α- and β-cells throughout life, including adult β-cells [7].
Functional roles in gene regulation	Activates β-cell genes. Synergizes with PDX1 and NeuroD1 to drive insulin transcription and regulates key β-cell identity genes (*Slc2a2*, *Nkx6.1*) [31].	Critical during development and directly activates insulin and glucagon transcription in embryonic endocrine cells. Binds to the key maturation gene PDX1 to promote β-cell differentiation [8]. Human MafB represses non-β-cell hormones (e.g., gastrin) in β-cells.
Involvement in diabetes	MafA promoter variants (reducing thymic MafA) are linked to higher T1D susceptibility [37]. Low MafA in human islets correlates with upregulation of innate immunity (*IFNAR1*) and T1D susceptibility (*IFIH1*) genes [38]. MafA is downregulated by glucotoxicity, as (db/db) mice lose MafA as c-Jun rises, leading to impaired insulin promoter activity [39]. In human pancreatic islets from patients with T2D, significant reduction in MafA observed in β-cells [40].	MafB deficiency in human β-cells causes loss of insulin expression and aberrant expression of other hormones (gastrin, PPY, SST). A subset of T2D β-cells expresses gastrin and SST [41]. MafB might have a role in macrophage infiltration of pancreatic islets, leading to insulitis in T2D [42].

**Table 2:** Summary of studies on mice and human cell line models detailing the role of MafB in islet development and β-cell function. N/A: Not necessary.

Model Description	Species	MafB Status	GSIS Outcome	Islet Architecture	Key Findings	Reference
*MafB*^*−*/*−*^ mouse embryos	Mouse	Knockout	N/A	Reduced number of insulin+ and glucagon+ cells. Endocrine cells count unchanged.	Late onset of insulin+ cells. β-cells lacking insulin lose *PDX1*, *NKX6.1*, and *GLUT2*.	[10]
*MafB^−^*^/*−*^ mice	Mouse	Knockout	N/A	N/A	MafB activates genes involved in mature endocrine function (*Slc2a2*, *Slc30a8*, *Camk2b*, *Nnat*). β-cell differentiation markers (*Pax6*, *NeuroD1*, *Pax4*, *Isl1*) unaffected in MafB^−/−^. MafA is required to sustain β-cell function.	[7]
MafB deficient (*kr^ENU^/kr^ENU^*) mutant mouse embryos	Mouse	Loss-of-function mutant	N/A	Reduced number of insulin+ and glucagon+ cells. Endocrine cells count unchanged.	MafB can activate insulin and glucagon gene expressions. MafB deficiency does not affect endocrine specification.	[27]
Endocrine-specific MafB KO (*Mafb^∆Endo^*) and tamoxifen-dependent MafB KO (*Mafb^∆TAM^*) (MafB deleted after pancreas maturation)	Mouse	Conditional knockout	N/A	Reduced number of insulin+ and glucagon+ cells at postnatal day 0, but insulin+ cell population recovered in 8 weeks in *Mafb^∆Endo^* mice. *Mafb^∆TAM^* mice showed reduced glucagon+ cells and glucagon content without affecting β-cells.	MafB is a key regulator of glucagon expression in α-cells.	[43]
Tamoxifen-inducible pancreatic MafB deletion on *MafA*^–/–^ background (A0B^*Δpanc*^ model)	Mouse	Conditional knockout (postnatal)	Impaired (glucose intolerance in intraperitoneal glucose tolerance test).	Decreased insulin+ cells and impaired islet architecture. β-cell dedifferentiation observed.	Impaired glucose tolerance, high urine glucose level, and renal lesions. Dedifferentiation markers (*Slc5a10* and *Cck*) upregulated; β-cell markers (*Slc2a2* and *Ucn3*) downregulated.	[40,44]
Pancreas-wide MafB mutant (*MafB^∆panc^*) and pancreas-wide MafA/MafB mutant (*MafAB^∆panc^*)	Mouse	Conditional knockout	Slightly elevated insulin secretion at 2.8 mM glucose in *MafB^∆panc^* at 2 weeks old. No changes for 20 mM glucose challenge. GSIS indistinguishable between wildtype and *MafB^∆panc^* at 8 weeks old.	Insulin+ cell production delayed in *MafB^∆panc^* until MafA comprehensively expressed.	β-cell activity delayed in *MafB^∆panc^* until MafA expressed. Authors propose MafA compensates for absence of MafB in *MafB^∆panc^* mice, supported by the death of *MafAB^∆panc^* after birth due to hyperglycemia.	[32]
Human EndoC-βH1 β-cell line, siRNA knockdown of MafB and MafA	Human EndoC-βH1 β-cell line	Knockdown	Reduced insulin secretion in MafA and MafB knockdown cells.	N/A	Impaired GSIS. Downregulation of exocytosis genes (*STX1A*, *SYT7*, *STXBP1*).	[29]
Human EndoC-βH1 and EndoC-βH2 β-cell lines, lentiviral knockdown of MafB	Human EndoC-βH1 and βH2 β-cell lines	Knockdown	N/A		MafB knockdown elevated non-β-cell markers *GAST*, *SST* and *PPY* mRNA and reduced *INS* mRNA.	[41]
CRISPR/Cas9-mediated MafB knockout in hPSC-derived β-like cells	Human ESC and iPSC lines	Knockout	No C-peptide secretion in transplanted knockout β-like cells.	Transplanted knockout grafts had elevated levels of PDX1+ cells, low levels NKX6.1+ cells and no C-peptide+ cells.	MafB knockout has normal pancreatic differentiation up to progenitor stage but favors somatostatin+ and pancreatic polypeptide+ cells at the expense of insulin and glucagon producing cells.	[28]

## 4. Potential Role of MafB in β-Cell Dysfunction and Diabetes

Given MafB’s developmental importance discussed in the sections above, it is evident that its loss or insufficiency may drive diabetes pathogenesis. T2D is characterized by peripheral insulin resistance and β-cell dysfunction, with the latter presenting in the form of β-cell loss and a loss of β-cell identity. Various lines of evidence link reduced MafB expression to β-cell dysfunction, and thus, MafB may contribute to diabetes pathogenesis via loss of β-cell identity and function. Notably, human islets from T2D patients exhibit reduced expression of MafB (along with MafA, NKX6.1, and PDX1) at the mRNA and protein levels [13,41]. Studies showed that loss of MafB disrupts β-cell identity by promoting β-cell dedifferentiation. Deng et al. demonstrated that deletion of *MafB* under *MafA*-deficient conditions (*A0B^Δpanc^*) in an adult diabetic mice model leads to β-cell dedifferentiation evidenced by upregulation of dedifferentiation markers, such as *Slc5a10* and *Cck*, and downregulation of mature β-cell markers, such as *Slc2a2* and *Unc3* [44]. This suggests a reversion of β-cells to an immature state due to loss of MafB, which may be attributed to diabetes pathogenesis.

In humans, studies on stem-cell-derived β-cells have revealed that MafB plays a critical role in insulin production (by preserving mature beta-cell identity) and suppression of non-β-cell hormone expression [28]. Silencing MafB in the human β-cell line EndoC-βH2 showed increased production of gastrin^+^ (GAST^+^) cells, a phenotype resembling changes that occur under chronic hyperglycemia and obesity [41]. This phenotype was also observed in human T2D β-cells with reduced MafB expression [41]. Aberrant expression of the fetal islet hormone gastrin in a subset of β-cells of T2D donors is a phenomenon rarely seen in healthy adult β-cells [45,46]. ChIP-Seq on human islets confirmed that MafB acts as a direct repressor of the *GAST* gene. Thus, when there is a loss of MafB in metabolically stressed β-cells, gastrin expression is reactivated, perhaps reverting β-cells into a fetal gene program [41]. Additionally, β-cells in T2D have been observed to aberrantly produce the δ-cell hormone somatostatin (SST) and the γ-cell hormone pancreatic polypeptide (PPY) [41,47]. Lentivirus-mediated knockdown of MafB protein in EndoC-βH2 cells elevated mRNA levels of *GAST*, *SST* and *PPY* [41]. Comparable results were observed in MafB-deficient stem-cell-derived β-like cells, wherein cells showed reduced expression of insulin while expression of *SST* and *PPY* were greatly increased [28]. Taken together, these results suggest that β-cells transdifferentiating into poly-hormonal cells under T2D conditions may coincide with the loss of MafB and may contribute to disease progression. Cha et al. proposed that the high sensitivity of MafB (and MafA) to metabolic stressors, relative to other islet-enriched transcription factors, leads to their compromised expression under conditions of obesity and insulin resistance, which leads to β-cell dysfunction and impaired cell identity due to the induction of GAST^+^ cell-associated gene products. The authors suggest this cascade of events represents an early stage of cellular reprogramming that might precede β-cell dedifferentiation and T2D [41,48].

Although this review focuses primarily on the role of MafB in pancreatic β-cell biology, emerging evidence suggests that MafB may also influence islet function indirectly through immune-cell-mediated mechanisms. MafB plays a well characterized role in macrophage differentiation and polarization, processes that have been linked to islet inflammation and β-cell dysfunction in both T1D and T2D. Therefore, studies on the role of MafB in immune systems and macrophages may provide important insights into how the immune signaling pathways could influence β-cell survival and function within the islet environment. In a 2019 study, Singh et al. reported that *MafA*^−/−^/*MafB*^+*/*−^ mice exhibited elevated expression of pro-inflammatory genes in islets and developed peri-islet clusters of CD4^+^ T cells and B220^+^ B cells, hallmarks of autoimmune inflammation characteristic of T1D [42,49]. This immune infiltration was markedly more pronounced in *MafA*^−/−^/*MafB*^+*/*−^ animals than in *MafA*^−/−^, suggesting that the partial loss of MafB disrupts the immune balance [42]. This implies that MafB may play a protective role and contribute to maintaining immune tolerance in islets and that its partial loss may predispose the pancreas to autoimmune-like inflammation.

Healthy islets house resident macrophages that normally help maintain tissue homeostasis by promoting islet development and normal GSIS [50]. It is important to distinguish between MafB functions that are macrophage intrinsic and those that are β-cell intrinsic, as both cell types contribute to islet homeostasis. In macrophages, MafB has been observed to regulate the expression of genes involved in clearing apoptotic cells and maintaining self-tolerance. A study on *MafB*^−/−^ mice revealed that macrophages lacking MafB from fetal liver, spleen and peritoneal tissue (not islets) had impaired efferocytosis (dead-cell clearance) due to downregulated expression of complement component C1q subunits—*C1qa*, *C1qb*, and *C1qc*—while luciferase and ChIP assays revealed MafB as a direct target to the MARE sequences of the subunits [51]. C1q is essential for opsonizing (preparing a cell to be susceptible to phagocytosis) apoptotic debris, and its deficiency is known to render autoimmunity in humans and mice [52,53]. Consistent with this evidence, *MafB*^−/−^ deficient mice, under conditions in which apoptotic cells accumulate due to X-ray irradiation, presented increased autoantibodies and glomerulonephritis. These findings link MafB to prevention of immune responses by enabling macrophages to quickly remove dying cells [51]. Although a lack of T-cell tolerance to autoantigens leading to autoreactive β-cell destruction is considered the main mechanism of T1D, macrophages from T1D animal model, non-obese diabetic (NOD) mice have exhibited defective efferocytosis and an increase in apoptotic β-cells in the newborn pancreas. Apoptotic pancreatic cell accumulation can lead to increased necrosis and inflammation, along with a release of autoantigens [54,55]. While the macrophage- and β-cell-specific roles may intersect during inflammatory states in the islet microenvironment, the mechanisms underlying this crosstalk remain unknown. While the role of MafB in systemic macrophage efferocytosis is known, there is no direct evidence linking MafB loss to islet efferocytosis. Therefore, exploring their potential crosstalk in maintaining islet function in healthy individuals and investigating possible mechanisms that may associate MafB loss in macrophages to impaired clearance of apoptotic β-cells and pathogenesis or progression of diabetes is needed.

Although the complete pathogenesis of T2D remains unclear, recent evidence from T2D islets suggests that insulitis, dominated by macrophage infiltration, plays a role in disease progression [56,57,58]. MicroRNA-155 (miR-155) is a proposed candidate that may play a role in this mechanism. In a recent study, Zhang et al. observed that inhibiting miR-155 could ameliorate high-fat-diet (HFD)-induced T2D pathogenesis in mice. The authors reported lentivirus-mediated miR-155 suppression improved GSIS and reduced insulin resistance and glucose intolerance in HFD feeding and db/db type-2 diabetic mice. They also observed that miR-155 levels were increased in islet-resident macrophages within the islets of T2D mice. Luciferase assays confirmed that miR-155-mediated effects on islets were due to its direct targeting of the key β-cell transcription factor PDX1 [58]. Interestingly, miR-155 is also a known suppressor of MafB in macrophages. Studies in bone marrow-derived macrophages have shown that miR-155 directly binds to the 3′ UTR region of *MafB*, thereby reducing its expression and pushing macrophages towards a heightened pro-inflammatory M1 state. Therefore, the enforced expression of miR-155 lowers MafB expression and could skew macrophages to produce more inflammatory mediators [59]. Thus, in T2D, a feed-forward loop might exist wherein metabolic/inflammatory signals can induce macrophage miR-155, which can downregulate MafB, and push islet macrophages to a pro-inflammatory state. However, direct evidence for this mechanism in islet-resident macrophages is currently lacking, and therefore, at present, this model can only be considered a plausible hypothesis. Future studies can further explore the miR-155– MafB regulatory axis and its impact on islet biology. For example, single-cell RNA sequencing of human islets from T2D and non-diabetic controls could be used to determine whether miR-155 expression correlates with MafB suppression in islet macrophage populations. Another direction of study can include macrophage-specific genetic manipulation of MafB in HFD mouse models and observing whether GSIS, β-cell stress markers, and macrophage polarization are affected in vivo. Thirdly, co-culture systems combining human islets with macrophages could be used to evaluate whether inhibition of miR-155 restores MafB expression and reduces inflammatory cytokine production while preserving β-cell function. Finally, spatial transcriptomic approaches may help determine whether specific macrophage activation states spatially correlate with β-cell stress or dedifferentiation signatures within the islet.

Additionally, more evidence of the effect of MafB in diabetes pathogenesis is seen in a study wherein bulk RNA-seq analysis on circulating peripheral blood mononuclear cells (PBMCs) of T2D patients showed increased levels of MafB in PBMCs [58]. In another study, qPCR analysis of PBMCs revealed increased MafB levels in T2D patients and was correlated with diabetic nephropathy [59]. The difference in results could be attributed to donor heterogeneity, cell type differences, and disease duration and progressions. Since different patterns of MafB expression are observed in different cell types, continued integration of multi-omics datasets from different tissues will be important for clarifying the precise patterns of MafB regulation in different tissues in diabetic individuals. Key studies that published evidence of the potential of MafB dysregulation in diabetes is presented in Table 3.

**Figure 3:**
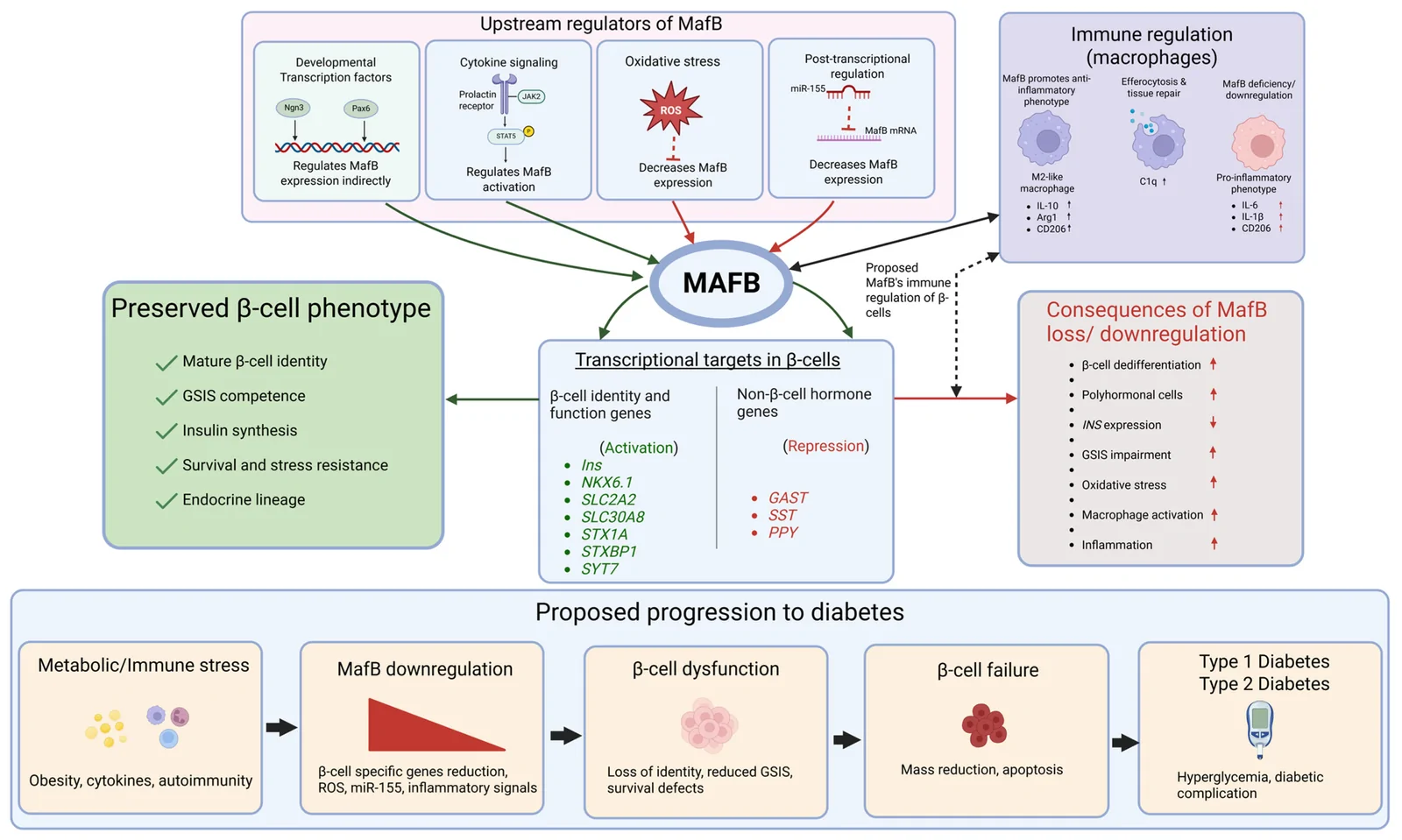
Proposed roles of MafB in β-cell function and dysfunction and possible mechanisms of its contribution to the pathogenesis of diabetes. Intrinsically in β-cells, impaired MafB results in loss of insulin expression and dysregulation of GSIS, while *SST*, *PPY* and *GAST* expressions are increased. MafB could also regulate β-cell function through immune-mediated pathways whose impairment results in β-cell dysfunction. Proposed mechanism of the contribution of MafB to the pathogenesis of diabetes is also illustrated. Created in BioRender. Al-Siddiqi, H. (2026) https://BioRender.com/bblq4i8 (accessed on 10 July 2026).

Collectively, these findings position MafB as a potential integrator of β-cell identity and islet immune homeostasis, whose loss could drive β-cell dysfunction through coordinated effects on cellular identity and hormone and islet immune regulation, thereby contributing to the pathogenesis of diabetes. Although further studies of MafB-dependent regulatory networks in β-cells and pancreatic health will be essential to define its precise role in diabetes pathogenesis, the unique position of MafB linking β-cell identity and immune-mediated islet dysfunction underscores its potential value as a translational target for preserving β-cell function in diabetes. A summary of the potential effects of impaired MafB on β-cells and diabetes pathogenesis is illustrated in Figure 3.

## 5. Potential Translational Application and Future Research Directions

A growing body of evidence linking MafB to β-cell differentiation, identity, and maintenance and the protective role it plays against chronic oxidative and metabolic stress highlights its potential relevance as a biomarker or molecular target in diabetes research. The possible translational applications of MafB in these contexts are outlined below.

(1) MafB expression in β-cell replacement

Studies on human stem-cell models have shown that MafB is required for the development of mature β-cells at the final stages of β-cell differentiation, as hPSCs lacking MafB failed to generate mature insulin-producing β-cells [28]. However, reexpression of MafB was able to rescue insulin production and secretion, reinforcing its key role in β-cell function [28] As a potentially unlimited source of β-cells of autogenic origin, stem cells can reduce dependence on organ donors and its associated complications for the treatment of diabetes. This aspect of stem cells has been explored in several clinical trials in recent years [62,63]. A common pitfall for the current differentiation protocols is that the resultant β-cells show relatively lower GSIS function (~5 times lower) as compared to cadaveric islets [35]. Incremental knowledge of β-cell development and enhanced differentiation protocols have narrowed the gap between hPSC-derived β-cells and human islets [64,65]. One proposed way to improve the yield and function of β-cells might be through the timely and apt expression of MafB in the differentiation process. Strategies to induce MafB expression during the pancreatic progenitor and endocrine induction phase of stem-cell-derived β-cell differentiation protocols could improve the yield and functionality of β-like cells. This could be achieved by screening for small molecules or growth factors that enhance MafB expression in β-cell differentiation. Furthermore, the timely delivery of transcriptional activators or epigenetic modulators could be utilized to promote MafB expression.

MafB is currently not known to be targeted by any drugs or small molecules. However, a few pharmacological strategies could indirectly preserve or revive its function in diabetic β-cells. Animal studies have revealed that MafB directly activates downstream genes responsible for the expression of the transcription factor PDX1 and the insulin gene promoter [28]. Theoretically, if loss of MafB leads to a decline in PDX1 and insulin levels, perhaps pharmacological agonists of alternative pathways such as GLP1R could be utilized to upregulate these targets and compensate for the deficit. A potential target for study is incretin-based therapies. The gene *PPP*_1_*R*_1_*A* (Protein Phosphatase 1 Regulatory Subunit 1A) has recently been identified as a novel MafA/MafB target gene that engages in GLP1R/PKA signaling, increasing mitochondrial coupling efficiency, and amplifying GSIS in β-cells. The authors suggested that reduced *PPP*_1_*R*_1_*A* expression may lead to T2D by reducing responsiveness to GLP1. Therefore, this pathway could be targeted in T2D conditions to restore β-cell function in the absence of MafB [66]. A case can also be made for targeting genes upstream of MafB to induce/improve MafB expression. One such example can be derived from a study that examined the prolactin receptor gene (PRLR) in gestational diabetes. Loss of the PRLR gene in β-cells resulted in gestational diabetes, and the authors observed that the PRLR gene is required for the transient expression of MafB within a subset of β-cells during pregnancy. This raises the possibility of activating prolactin-mediated Jak2/Stat5 pathways to upregulate MafB expression and promote β-cell proliferation and function. Although this study was focused on gestational diabetes, further studies can be done to explore this pathway and its relevance to treatment of T2D. Any intervention to boost MafB expression needs careful consideration and calibration since excessive MafB could have potential unintended side-effects, such as driving the cells towards α/β dual identity since MafB is crucial to the development of both cell types.

Taken together, the studies above open opportunities to boost MafB expression in β-cells to enhance insulin production and secretion, which can potentially form a key additive translational approach to treat diabetes along with the current treatment strategies. Each of the approaches discussed are still largely theoretical for MafB specifically. We anticipate that these strategies can be evaluated in diabetic models to see if β-cell function improves. However, the models used to study the translational effects of MafB need to be carefully considered. Since adult murine β-cells largely lack MafB expression, whereas adult human β-cells maintain substantial MafB expression, therapeutic responses to MafB modulation are unlikely to be directly comparable between species. Rodent preclinical models of MafB overexpression are likely to show limited phenotypic readouts in adult β-cells; therefore, their value would be mainly for safety/off target screening. For direct effects of MafB in human β-cells, future studies should prioritize human islets, stem-cell-derived β-cells, and humanized mouse models. We anticipate that MafB upregulation in human β-cells may preserve β-cell identity, maintain insulin gene expression, improve GSIS, reduce dedifferentiation, and improve responses to metabolic and oxidative stress. However, these proposed outcomes remain hypotheses requiring rigorous experimental validation. It will also be important to ensure that manipulating MafB does not have off-target effects in other tissues where MafB is expressed. MafB is not restricted to pancreatic islets, as it performs essential, dosage-sensitive roles in at least two other organ systems, and any strategy aimed at pharmacologically raising MafB activity for β-cell preservation would need to account for this. The best-characterized role for MafB is within the macrophage lineage [67]. MafB is a master regulator of macrophage differentiation and maturation, where it promotes acquisition of the mature macrophage phenotype while restricting self-renewal [68]. Since macrophages contribute to tissue homeostasis, host defense, and inflammatory responses, systemic modulation of MafB could influence immune function beyond the islet, potentially altering inflammatory responses in other organs or modifying susceptibility to infections. Moreover, MafB is also known to play essential roles in renal development, as MafB-deficient mice demonstrated impaired kidney development with abnormal podocyte differentiation, along with tubular apoptosis [69]. Although these observations primarily reflect developmental loss of MafB rather than therapeutic overexpression, they indicate that sustained systemic modulation of MafB should be carefully evaluated for potential renal side-effects. Another well-established function of MafB is the regulation of osteoclast differentiation. During osteoclastogenesis, MafB acts as a negative regulator of RANKL-induced differentiation by suppressing the transcriptional activity of NFATc1, c-Fos, and MitF, thereby limiting osteoclast maturation while preserving macrophage function [70] Taken together, these findings suggest that future therapeutic strategies should prioritize β-cell-selective MafB modulation rather than systemic activation. Approaches including β-cell-specific viral vectors, targeted lipid nanoparticles, cell-selective epigenetic editing, or glucose-responsive gene expression systems may maximize therapeutic benefit while minimizing undesirable effects in macrophages, kidney, bone, and other MAFB-expressing tissues. Comprehensive evaluation of tissue specificity and long-term safety will therefore be essential before MAFB-directed therapies can be translated into clinical practice. Consequently, prolonged systemic activation of MAFB could theoretically influence bone remodeling and skeletal homeostasis.

In summary, targeting MafB offers a novel avenue in diabetes research wherein restoring or enhancing MafB activity through gene therapy or drugs could help restore insulin production and secretion by strengthening β-cell identity.

(2) Oxidative stress modulation

Oxidative stress is a major driver of β-cell dysfunction in diabetes. Evidence indicated that MafB is highly susceptible to oxidative stress in β-cells, with reduced expression reported under reactive oxygen species (ROS)-rich conditions leading to the loss of β-cell identity [71]. In a diabetic mouse model, overexpression of the antioxidant enzyme GPX1 restored nuclear MafA and improved β-cell function [13]. Although direct evidence on MafB is unavailable, these findings support the hypothesis that antioxidant treatments might protect MafB against oxidative stress in diabetic β-cells. Future studies could assess whether known β-cell-targeted antioxidants or ROS scavengers stabilize MafB or preserve β-cell identity and function under hyperglycemic or oxidative stress conditions.

(3) Targeting MafB-related immune regulation

As discussed in earlier sections, MafB’s role is not limited to β-cells, as it is also expressed in islet-resident and infiltrating macrophages, where it promotes an anti-inflammatory phenotype. Loss of MafB in macrophages moves them to a more inflammatory state, contributing to islet inflammation and β-cell dysfunction in diabetic contexts. Therapeutic strategies designed to boost MafB in macrophages could help restore immune balance in the islet microenvironment. For example, blocking miR-155 (which suppresses MafB) in a therapeutic setting could improve MafB expression and possibly reverse the inflammatory effects of macrophages [59].

(4) Emerging evidence of MafB expression in PBMCs as an early biomarker for T2D

Emerging evidence suggests that MafB expression in peripheral blood mononuclear cells (PBMCs) may be associated with metabolic dysfunction and could potentially serve as an early indicator of diabetes risk. In a recent study, PBMCs from patients with T2D showed higher expression of MafB was associated with insulin resistance (HOMA-IR) and inflammatory signaling, including tumor necrosis factor-α (TNF-α). A multivariate logistic regression analysis further indicated that MafB in PBMCs has a predictive value for T2D, supporting its potential to serve as a biomarker for early identification of at-risk individuals [61]. Zhang et al. suggest that MafB, fasting blood glucose and HbA1c as covariates outperform any of the standard individual variables in diagnosing T2D, therefore highlighting the value of MafB in assisting the diagnosis of T2D. MafB, however, is broadly expressed in myeloid lineage cells, including monocytes and macrophages, and its expression in the PBMCs may therefore reflect systemic immune activation or inflammatory states rather than β-cell-specific pathology. Therefore, changes in PBMC MafB levels need to be interpreted cautiously and cannot currently be assumed to directly represent MafB dynamics within pancreatic islets. Several factors may influence PBMC transcriptional profiles and thus confound interpretation of MafB expression levels. These may include body mass index (BMI), acute or chronic infections, smoking status, medications commonly used in metabolic disease management (such as metformin and GLP-1 receptor antagonists), and other comorbid inflammatory conditions. Future studies evaluating MafB as a circulating biomarker will therefore require careful consideration of these variables. Zhang et al. also highlighted key limitations to this study wherein the sample size for this study was relatively small, the study could not determine a causal relationship between MafB and T2D, the datasets used for bulk RNAseq analysis had different ages and sample sizes that could confound the results, as well as the relationship between MafB and chronic inflammation required further study of the underlying molecular mechanism [61]. Similarly, in another study, MafB (among other markers) levels in PBMCs have been implicated as an indicator of diabetic nephropathy severity and can distinguish diabetic nephropathy from normoalbuminuria [60].

Despite growing interest in MafB as a potential circulating marker, several limitations currently restrict its translational application. Firstly, PBMC MafB expression may not accurately reflect MafB regulation within pancreatic islets, as they represent distinct cellular environments with different regulatory mechanisms. Establishing a mechanistic link between β-cells and immune cell MafB expression remains an important research priority. Future validation efforts should ideally include paired PBMC and pancreatic islet samples obtained from the same donors, which could allow for direct comparison of MafB expression patterns across cell types. Longitudinal cohort studies will also be required to determine whether PBMC MafB levels predict future metabolic decline or diabetes onset. Moreover, vigorous evaluation of receiver operating characteristic (ROC) analysis and area-under-the curve (AUC) measurements and replication in an independent external cohort will be necessary before MafB can be considered a dependable biomarker candidate. Until such validation studies are conducted, PBMC MafB expression should be viewed as a promising but preliminary signal rather than a clinically significant marker.

Although MafB has emerged as an important regulator of pancreatic endocrine development and β-cell function, its direct translational application remains largely exploratory. Current evidence supporting the roles of MafB as a biomarker or therapeutic target is limited, and most studies have focused on transcriptional regulation or immune-cell biology rather than direct pharmacological modulation. Therefore, it is crucial to distinguish between short-term research directions and longer-term translational possibilities.

## 6. Conclusions

In summary, MafB plays an important regulatory role in the development, maintenance, and survival of pancreatic β-cells. Its roles extend from embryonic pancreatic development through the maintenance of adult β-cell identity and function as well as immune homeostasis. MafB works with key β-cell transcription factors to activate insulin transcription and preserves β-cell activity and identity by repressing non-β-cell hormone’s expression, such as gastrin, somatostatin, and pancreatic polypeptide. Loss of MafB in β-cells has been shown to have impaired insulin production and secretion, dedifferentiation of β-cells and transdifferentiation under metabolic stress, which are key markers of diabetic pathology. MafB’s dual role to regulate β-cell identity and function and promote anti-inflammatory macrophage polarization may identify it as a unique metabolic and immunological regulatory factor. This functional duality highlights its potential to serve as a therapeutic target for preserving β-cell function and survival while simultaneously attenuating the pro-inflammatory signaling that drives diabetes pathogenesis. To clearly distinguish between established findings and emerging hypotheses, it is critical to note that the role of MafB in pancreatic β-cell development and endocrine lineage specification is strongly supported by genetic and functional studies in both mouse and human systems. Approaches to boost MafB, whether by activating its expression in failing β-cells by small molecules or drugs or modulating its pathways to enhance insulin production, may help regenerate β-cell mass or function. In addition, several proposed links between MafB and immune regulation and the circulating biomarkers discussed in this review are supported primarily by studies in macrophages, PBMCs and other indirect experimental systems. Although these studies provide interesting insights into potential systemic roles of MafB, they require rigorous further validation in pancreatic islets and in vivo models. Further research of MafB regulatory circuits, interaction networks, and stress-response pathways is essential to develop new targeted therapies aimed at preserving and restoring β-cell function in diabetes. As β-cell replacement strategies and in vivo reprogramming therapies advance, MafB is poised to play a significant role in revealing new mechanisms of disease progression and providing opportunities to leverage its potential in understanding diabetes. However, critical knowledge gaps remain in our current understanding of its transcriptional network, gene upstream regulation, and responsiveness to pharmacological interventions. Future studies should (1) investigate MafB regulation in human diabetes, (2) test MafB-based therapies in animal and hPSC models, and (3) examine how MafB interacts with other β-cell factors under stress. Filling these gaps will be essential for translating MafB biology into diabetes treatment.

## Figures and Tables

**Table 3:** Summary of the published evidence of potential MafB dysregulation in diabetes.

Study Cohort	Methods	Key Findings	Key Caveats	Reference
Human T2D vs. control islet β-cells	Immunohistochemistry and qPCR	Reduction in MafB expression	Small donor number (*n* = 8)	[13]
Human T2D vs. control islets	Immunohistochemistry	Enrichment of GAST-positive cells in T2D islets deficient in MafB	Small donor number (*n* = 8 control, *n* = 7 diabetic)	[41]
PBMC samples from T2D, control and diabetic nephropathy patients	qPCR	MafB expression increased in patients with diabetic nephropathy	Small sample size, possible interference from medications. Emerging evidence of link between MafB and T2D, requires further validation.	[60]
PBMC samples from T2D, control, and impaired glucose tolerance individuals	qPCR, Western blotting and single-cell RNA seq	High MafB is associated with insulin resistance and chronic inflammation in T2D patients	Increase in MafB expression is mainly observed in nonclassical monocytes. Emerging evidence of link between MafB and T2D, requires further validation.	[61]

## Data Availability

No new data were created or analyzed in this study. Data sharing is not applicable.

## Abbreviations

The following abbreviations are used in this manuscript:

MAF	Musculoaponeurotic Fibrosarcoma
MafB	Musculoaponeurotic Fibrosarcoma B
IDF	International Diabetes Federation
T1D	Type 1 Diabetes
T2D	Type 2 Diabetes
MARE	MAF Recognition Element
bZIP	Basic Leucine Zipper Domain
TRE	TPA Responsive Element
CRE	cAMP Responsive Element
MLL3/4	Methyltransferase Coregulator Complexes Mixed-Lineage Leukemia 3 and 4
GSIS	Glucose Stimulated Insulin Secretion
MODY	Mature Onset Diabetes of the Young
Ngn3	Neurogenin-3
hPSC	Human Pluripotent Stem Cell
NOD	Non-obese Diabetic
GAST	Gastrin
SST	Somatostatin
PPY	Pancreatic Polypeptide
miR-155	MicroRNA-155
HFD	High Fat Diet
*PPP*_1_*R*_1_*A*	Protein Phosphatase 1 Regulatory Subunit 1A
PBMC	Peripheral Blood Mononuclear Cell
ROC	Receiver Operatic Characteristic
AUC	Area-Under-the-Curve
TNF-α	Tumor Necrosis Factor-α
ROS	Reactive Oxygen Species
